# *Nannochloropsis oceania*-derived defatted meal as an alternative to fishmeal in Atlantic salmon feeds

**DOI:** 10.1371/journal.pone.0179907

**Published:** 2017-07-13

**Authors:** Mette Sørensen, Yangyang Gong, Fridrik Bjarnason, Ghana K. Vasanth, Dalia Dahle, Mark Huntley, Viswanath Kiron

**Affiliations:** 1 Faculty of Biosciences and Aquaculture, Nord University, Bodø, Norway; 2 Key Laboratory of East China Sea and Oceanic Fishery Resources Exploitation and Utilization, Ministry of Agriculture, East China Sea Fisheries Research Institute, Chinese Academy of Fishery Sciences, Shanghai, China; 3 Department of Biological and Environmental Engineering, Cornell University, Ithaca, NY, United States of America; 4 Marine Laboratory, Nicholas School of the Environment, Duke University, Beaufort, NC, United States of America; Universidade de Vigo, SPAIN

## Abstract

Defatted microalgal biomass derived from biorefinery can be potential feed ingredients for carnivorous fish. The present study investigated the growth, feed intake:gain and health parameters in Atlantic salmon fed for 84 days with defatted *Nannochloropsis oceania* as a fishmeal replacer. Fish fed feeds containing the algal biomass (at 10 and 20% inclusion, alga groups) were compared with groups that consumed alga-devoid feeds (control group). The fish that received 20% alga tended to have reduced weight gain and specific growth rate. Condition factor, feed conversion ratio and feed intake of this fish group were significantly different when compared with the control group. Hepatosomatic and viscerosomatic indices, whole body and fillet proximate composition were not affected by the dietary treatments. Digestibility of dry matter, protein, lipid, ash and energy, as well as retention of lipid and energy of the fish that received feed with 20% alga meal were also significantly different from those of the control group. Serum superoxide dismutase activity of the 10% alga-fed fish was significantly higher compared with the control fish. Although alga feeding did not cause any distal intestinal inflammation, the intestinal proteins that were altered upon feeding 20% algal meal might be pointing to systemic physiological disturbances. In conclusion, feeds with 20% alga had a negative effect on feed intake, FCR, lipid and energy retention and health of the fish. The defatted *Nannochloropsis oceania* can be used at modest inclusion levels, around 10%, without negative effects on the performance of Atlantic salmon.

## Introduction

Marine microalgae are unicellular organisms, and they are rich in high-quality protein, essential amino acids, polyunsaturated fatty acids, sugars, polysaccharides, vitamins, minerals and pigments [1]. Certain microalgal varieties are already marketed for human consumption. However, microalgae biomass is hardly exploited commercially as aquafeed components, primarily due to their unavailability in large volumes and high price of the marketed products. Researchers are developing strains that contain more lipids, nutrients as well as bioactive compounds [2], and “biocrude” oil, and residual protein-rich fractions are co-products of cultivated microalgae [3]. Thus, the biofuel and the defatted biomass that is rich in protein will be available in large amounts in the near future.

*Nannochloropsis* is a candidate that is exploited for biofuel production because of their high lipid content [4]. The lipid content may vary from 1 to 40% of dry matter (DM) in certain strains, and under special culture conditions the level can go up to 85% [5]. In *Nannochloropsis*, eicosapentaenoic acid (EPA) is the dominant fatty acid [6], and this characteristic makes the microalga a potential partial fish oil replacer in fish feeds [7]. Feeding salmon with plant oils can reduce the levels of EPA and DHA in the fish flesh [8]. Atlantic salmon can endogenously convert long-chain PUFAs from their dietary sources, due to the presence of desaturase and elongase genes [9–11]. The presence of fatty acyl elongase *elovl2* helps the fish to elongate C20 and C22 fatty acids [11], suggesting the ability of salmon to utilize the PUFAs derived from microalgal lipids. On the other hand, the protein-rich biomass of *Nannochloropsis* can be a potential fishmeal replacer because of its nutrient content. Many research groups have reported the suitability of defatted microalgae as feed ingredients in the feeds of aquatic animals. Kiron et al. [12] showed that 10% of fishmeal protein in the feeds for Atlantic salmon post smolts can be replaced with the protein from defatted *Nanofrustulum*; without negatively affecting the growth performance, feed performance and body composition of the fish. Patterson and Gatlin III [13] also reported that up to 10% crude protein from fishmeal and soy protein concentrate in the feeds for red drum could be replaced with lipid-extracted algae meal (derived from *Navicula* sp., *Chlorella* sp. and *Nannochloropsis salina*); without negatively affecting the growth, feed utilization, protein and energy retention of the fish.

Although the potential of defatted biomass to support the growth of aquatic animals was demonstrated in earlier experiments, each alga strain needs to be tested on each target species. The results from our earlier experiments on Atlantic salmon have indicated that the digestibilities of protein from *Desmodesmus* and *Nannochloropsis* were not different [14, 15]. However, experiments on a mammalian carnivore model, mink, have pointed out that the digestibility of protein from different microalgae vary widely [16]. Digestibility coefficient estimation is however only the first step to evaluate the bioavailability of nutrients for growth and therefore, the observations need to be verified through long-term feeding experiments [17]. It is also important to assess the effects of the tested feed ingredients on the health of the animals.

In the present study, the effect of the replacement of fishmeal with *Nannochloropsis oceania* (*N*. *oceania*) biomass in the feeds of Atlantic salmon was evaluated on the growth performance, feed utilization and intestinal health of the fish. An 84-day feeding trial was conducted to examine the growth and feed performance, antioxidant status, expression of genes and proteins, and micromorphology of the distal intestine of Atlantic salmon.

## Materials and methods

### Experimental design and feeds

The study, approved by the National Animal Research Authority (FDU: Forsøksdyrutvalget ID—5887) in Norway, consisted of three groups: a control group (1C- offered control feed), and 2 algal groups [offered feed with 10% (1L) and 20% (1H) alga meal]. The algal meal is the biomass from *N*. *oceania*, a product obtained after biofuel extraction (Cellana, San Diego, USA). Chemical composition of the algae biomass is presented in S1 Table. The content of elements, amino acids, fatty acid composition, neutral detergent fiber (NDF) and acid detergent fiber (ADF) was reported by Gatrell et al. [18].

Ingredients and chemical composition of the feeds are presented in Table 1. The control feed was based on fishmeal while algal biomass replaced 100 and 200 g fishmeal. All other ingredients except fish oil were kept constant. Fish oil was reduced in the 1H feed to keep a constant crude protein:energy ratio. The extruded experimental feeds were produced by the Feed Technology Center (ForTek), Norwegian University of Life Sciences (NMBU), Ås, Norway. The extrusion equipment used for producing the feeds has been described earlier by Sørensen et al. [19, 20]. The feed ingredients were first mixed in a portable mixer (40L, Ide-Con AS, Norway), and then fed to the extruder using a Coperion Key-Tron feeder (Type T32, Coperion K-Tron International, New Jersey, USA). This feeder was calibrated to directly deliver the mixture, at an input rate of 54–55 kg mash/h, into the extruder barrel. The screw configuration was optimised to improve the mixing efficiency and feed quality, and for efficient utilisation of mechanical energy even at a lower feeding rate. Conditioning was initiated in the second section of the extruder barrel by adding both steam and water. The temperature profile for conditioning the two algal feeds in the five section extruder barrel was 39–40, 90–91, 121–123, 106–107, 66–67^°^C, respectively. The control feed was produced at slightly higher temperature ranges: 37–38, 99–105, 127–127, 109–113 and 82–91^°^C. The conditioned material was passed through four 2 mm dies, resulting in pellets with 2.0–2.1 mm diameter and 3.8–4.2 mm length. The operating pressures used for making the 1C, 1L and 1H feeds were 21, 25 and 31 bar, respectively, and torque fluctuated between 334–351, 315–366 and 342–381 Nm, respectively. Pellets were collected and conveyed pneumatically to an NMBU-FORBERG fluidized bed dryer (Forberg, Oslo, Norway) and dried to the final DM content of 936–942 g kg DM^-1^ (Table 1) in small experimental batch dryers (10 kW heater, 2550 m^3^h^-1^ fan capacity, keeping the product temperature < 55^°^C). The feeds were shipped to Nord University where they were stored in airtight containers at 4^°^C, until they were distributed to the feeders.

**Table 1:** Ingredients and proximate composition of the control and the microalga-containing feeds.

Ingredients (g/1000 g)	Experimental feeds		
	1C	1L	1H
Fish meal^1^	690	590	495
Algal meal^2^	0	100	200
Wheat^3^	120	120	120
Wheat gluten^4^	50	50	50
Fish oil^1^	135	135	130
Microingredients^5^	5	5	5
Marker^6^	0.1	0.1	0.1
Proximate composition (g/1000 g)			
Dry matter	942.4	942.4	935.6
In dry matter:			
Crude protein	564.0	535.3	501.2
Crude lipid	211.0	205.9	199.9
Ash	114.2	123.4	132.5
Energy (MJ/1000 g)	23.6	23.6	23.1

^1^ Nordsildmel AS, Bergen, Norway

^2^ Cellana LLC, Kona, Hawaii, USA

^3^ Felleskjøpet AS, Moss, Norway

^4^ Gluten Vital, Alimenta AS, Oslo, Norway

^5^ Vitamin and mineral mix is a proprietary formulation of Europharma, Leknes, Norway.

^6^ Yttrium, Metal Rare Earth Limited, Shenzhen, China

### Fish rearing facility, fish husbandry and feeding

The feeding experiment was carried out in a flow-through system at the Research Station, Nord University, Bodø, Norway. The circular fibreglass rearing tanks (800 l and 0.9 m deep) were custom made (A-plast, Skodje, Norway); they are slightly conical with top and bottom diameters of approximately 1 m and 0.9 m, respectively. The cone-shaped bottom with approximately 22 degrees slope ensures the efficient collection of faeces and left-over feeds. The tank design is provided in S1 Fig. Every automatic feeder (ArvoTec T-drum 2000 feeder) of the rearing system is equipped with a 1 g dosing drum, control cabinets and software (ArvoTec, Huutokoski, Finland). The left-over feeds were collected from the water drains in a 17 l tank-mounted solid waste collector (Aquatic Eco-Trap, Pentair Aquatic Eco-Systems®, Fl, USA).

Atlantic salmon (*Salmo salar*) post-smolts (Aquagen strain, Aquagen AS, Trondheim, Norway) were purchased from a commercial producer (Cermaq Norway AS, Hopen, Norway) and maintained at the research station on a commercial feed until they were used for the feeding trials.

The fish used for the experiment were of mean initial weight 215.4 ± 27.1 g. Fish in 6 replicate tanks (15 fish/tank; mean biomass 3232 g/tank) were allocated to one feed group. Seawater (33 g l^-1^ salinity) that was used for rearing the fish was pumped from 250 m depth in Saltenfjorden, filtered and aerated. The rearing water temperature, ranged from 6.7 to 7.1°C and the feeding period was 594.8 degree days. Oxygen saturation in the tanks, as measured at the outlet, was kept above 90%. A 24-h lighting regime was maintained in the rearing facility.

The experimental feeds were fed to fish during the experimental period of 84 days. The feeding procedure aimed at maximum voluntary feed intake by all groups of fish. Fish were fed two meals per day; first meal from 08.00–09.00 and second meal from 14.00–15.00. The left-over feeds were collected immediately after the two feeding sessions.

### Fish sampling

Fish handling and sampling procedures were in accordance with the protocols approved by the FDU. Before weighing and sampling, the fish were anaesthetised with MS-222 (Tricaine methane sulphonate; Argent Chemical Laboratories, Redmond, USA; 80 mg/l). The fish for sample collection at the start and at termination of the experiment were euthanized by a sharp blow to the head. Fish that were not removed for sampling at termination of the experiment were returned to the fish holding facility, to be used for other purposes, thereby adhering to the principle of reducing the number of fish sacrificed for the study.

Individual weight and length were taken at the start and end of the experiment. Initial samples for whole body and fillet chemical composition were obtained from 6 and 12 fish, respectively. For the end-of-the-study sampling, blood was drawn from the caudal vein of 3 fish/tank to assess the haematocrit values and to collect serum for enzyme assays. Thereafter, these fish were dissected and the visceral organs (without heart and kidney) and the liver were removed for calculating the viscerosomatic and hepatosomatic indices. The fillet of these 3 fish were collected, sealed in plastic bags and kept frozen at -40°C until they were used for analysing the fillet proximate composition (FPC). Distal intestinal samples intended for molecular studies were snap frozen in liquid nitrogen and transferred to a -80°C freezer. Histology samples of the distal intestine were rinsed with PBS and fixed in phosphate-buffered formaldehyde solution. Whole body of 6 fish from each tank were collected (after the term for faeces collection), sealed in plastic bags and frozen at -40°C to determine the whole body proximate composition (WBC).

Faecal samples were collected after the feeding trial, from the remaining 12 fish/tank. For determining the digestibility of the feeds, the fish were stripped two times (one week time interval between strippings), employing the procedure described by Austreng [21]. The samples from fish in one tank were pooled and kept frozen.

### Chemical analysis

The WBC of the initial samples were analysed individually (n = 6 fish), while those of the final samples were analysed after pooling the 6 fish from one tank (obtained at the end of the experiment). For the analysis of FPC of the initial samples, fillets from 2 fish (4 fillets) were pooled (n = 6). The FPC of the final samples were determined after pooling 6 fillets that were obtained from 3 fish from a tank (described previously).

Fish whole body and fillet samples were thawed and homogenized prior to chemical analysis (dry matter, ash, nitrogen, crude lipid) and energy determination. Faecal samples were freeze-dried prior to chemical analysis (dry matter, ash, nitrogen, crude lipid and yttrium) and energy determination. Faecal matter of fish from two tanks of the same group were pooled to secure enough material for the analysis.

Dry matter was determined by oven drying (105°C for 20 h) to constant weight (ISO 6496–1999), crude protein by Kjeldahl method (N × 6.25; Kjeldahl Auto System, Tecator Systems, Höganäs, Sweden; ISO 5983–1987), crude lipid by Soxhlet method with acid hydrolysis (Soxtec HT6, Tecator, Höganäs, Sweden; AOAC Method 954.02), ash by incineration in a muffle furnace at 540°C for 16 h (ISO 5984–2002), and energy by bomb calorimetry (IKA C200 bomb calorimeter, Staufen, Germany; ISO 9831–1998), yttrium by inductive coupled plasma mass spectroscopy (ICP-MS; performed at Eurofins, Moss, Norway; NS-EN ISO 11885). The proximate composition, energy and yttrium content of the samples were measured in duplicates.

### Antioxidant markers

The antioxidant status of the fish was evaluated by performing different assays, employing the serum aliquots. The total antioxidant capacity (TAC), catalase (CAT) activity and super oxide dismutase (SOD) activity were determined using kits from Cell Biolabs (STA-360, Cell Biolabs Inc., San Diego, CA, USA), Cayman Chemicals (707002, Cayman Chemicals, Ann Arbor, MI, USA), and Cell Biolabs (STA-340, Cell Biolabs Inc.), respectively. The protocols are described in our previous paper [22].

### Gene expression

The mRNA levels of antioxidant-related (*superoxide dismutase1*—*sod1*; *nuclear factor erythroid 2-related factor*—*nrf2*), gut mucosa-related (*immunoglobulin T*—*igt*), inflammation-related (*interleukin 1b*—*il1b*; *interleukin 10*—*il10*; *interleukin 17d*—*il17d*; *transforming growth factor beta—tgfb*), antimicrobial (*cathelicidin 1* and *2*—*cath1*, *cath2*) genes were assessed in this study.

The total RNA from the frozen tissues were extracted using E-Z 96 Total RNA Kit (OMEGA Bio-Tek Inc, USA) following the instructions from the manufacturer. To quantify the mRNA level of a particular gene, samples from 12 fish/group (2 from each tank) were considered. The list of primers of the genes [22–24] and the detailed protocol for the analysis [22] are described in our previous publications. List of primers of the differentially expressed and reference genes are presented in Table 2.

**Table 2:** List of primers for the differentially expressed genes and the reference genes.

Gene name	Sequence(5’-3’)	Amplicon size (bp)	PCR efficiency (%)	GenBank accession numbers
Target genes				
*sod1*	CCACGTCCATGCCTTTGGR-F	141	95.3	AY736282.1
	TCAGCTGCTGCAGTCACGTT-R			
*il17d*	CTTGTCTCCCTGGGCATACAG-F	201	112.7	EU689087.1
	CAATATGCCTCGGGTATGAACCT-R			
*igt*	CAACACTGACTGGAACAACAAGGT-F	97	107.7	GQ907004
	CGTCAGCGGTTCTGTTTTGGA-R			
Reference genes				
*ef1ab*	TGCCCCTCCAGGATGTCTAC-F	59	96	BG933853
	CACGGCCCACAGGTACTG-R			
*rpl13*	CGCTCCAAGCTCATCCTCTTCCC-F	79	96.4	BT048949.1
	CCATCTTGAGTTCCTCCTCAGTGC-R			
*rps29*	GGGTCATCAGCAGCTCTATTGG-F	167	94.5	BT043522.1
	AGTCCAGCTTAACAAAGCCGATG-R			
*ubi*	AGCTGGCCCAGAAGTACAACTGTG-F CCACAAAAAGCACCAAGCCAAC-R	162	92.7	AB036060.1

### Protein expression

The distal intestinal proteomes of the three groups (n = 6/group) of fish were analysed employing two-dimensional electrophoresis and liquid chromatography and tandem mass spectrometry (LC-MS/MS). The protein extraction and the 2-DE were carried out as described previously [24]. The LC-MS/MS work was undertaken at the Tromsø University Proteomics Platform, Tromsø, Norway. Gel analysis and protein identification were performed as detailed in our previous paper [22].

### Distal intestinal morphology

Approximately 5 μm sections were prepared from the distal intestinal samples of 6 fish per group. The sections were stained with Alician Blue-Periodic Acid Shiff’s reagent and the photomicrographs were prepared as described in our previous papers [22, 24].

### Calculations and statistical analysis

The apparent digestibility coefficients (ADCs) of DM, protein, lipid, ash and energy were calculated using the following equation [25]:

$$\mathrm{AD}C_{nutrient\;or\;energy}=\left[1-\left(\frac{Marker_{feed}\times Nutrient_{faeces}}{Marker_{faeces}\times Nutrient_{feed}}\right)\right]\times 100$$ (1)

$$\mathrm{AD}C_{drymatter}=\left[1-\left(\frac{Marker_{feed}}{Marker_{faeces}}\right)\right]\times 100$$ (2)

where *Marker*_*feed*_ and *Marker*_*faeces*_ are the contents of the marker (% dry matter) in the feed and faeces, respectively, and *Nutrient*_*feed*_ and *Nutrient*_*faeces*_ are the nutrient contents (% dry matter) in the feed and faeces.

Specific growth rate (SGR) and thermal unit growth coefficient (TGC) were calculated based on mean weights, employing the equations:

$$\mathrm{SGR}=[\frac{\left(ln\;W_{1}-lnW_{0}\right)}{t}]\times 100$$ (3)

$$\mathrm{TGC}=\frac{\left(W1^{1/3}-W0^{1/3}\right)\times 1000}{d^{o}}$$ (4)

W0 is the initial weight, W_1_ is the final weight, and t is the time (days), and d^o^ is the total number of degree days.

The organosomatic indices namely, hepatosomatic index (HSI), viscerosomatic index (VSI) and condition factor (CF) were calculated using the formulae:

$$\mathrm{HSI}=\left(\frac{Liver\;weight\;(g)}{Final\;body\;weight\;(g)}\right)\times 100$$ (5)

$$\mathrm{VSI}=\left(\frac{Viscera\;weight\;(g)}{Final\;body\;weight\;(g)}\right)\times 100$$ (6)

$$\mathrm{CF}=\frac{Body\;weight\;\left(g\right)}{Fish\;fork\;length^{3}\left(cm\right)}\times 1000$$ (7)

Feed conversion rate, FCR, was calculated using the formula:

$$\mathrm{FCR}=\frac{Dry\;matter\;feed\;intake\left(g\right)}{Weight\;gain\left(g\right)}$$ (8)

Retentions (Ret) of nitrogen (or energy) were calculated for each tank employing the following formula:

$$\mathrm{Ret}=\left(\frac{\left[\left(FB\times N_{f}\right)-\left(IB\times N_{i}\right)\right]}{\left(DM\;Feed\;intake\times N\;feed\right)}\right)\times 100$$ (9)

where IB and FB are the initial and final biomass and N is the concentration of the nitrogen (or energy) in fish (subscripts i and f represent initial and final samples, respectively) or feed.

Statistical analyses were carried out using Graphpad Prism 6 (Graphpad Software Inc., La Jolla, CA, USA). Normality and equal variance of the data were tested before performing one-way ANOVA. Tukey's multiple comparisons test was employed to detect the significant differences between the means of interest. Kruskal-Wallis test followed by Dunn's multiple comparisons test was employed in the case of non-parametric data, to understand the differences between the study groups. The differences between groups were considered significant at *P* < 0.05, and differences at 0.10 > P > 0.05 suggests a trend.

## Results

### Growth and feed performance

The fish had good health and growth during the experimental period; mortality was not recorded and the final weights of fish in the 3 groups were approximately twice that of their initial weights. Weight gain (P = 0.09) and SGR (P = 0.09) tended to differ among the feeding groups (Table 3). The fish fed the 20% alga-feed tended to have lower weights and SGR compared to that of the control fish. The FI of the 1H group was significantly higher compared to that of the 1C group, and the FCR of the 1H group was significantly higher than those of the 1L and 1C groups. The TGC and PER of the alga-fed fish were lower (P>0.05) than the values of the 1C group.

**Table 3:** Survival, growth and feed utilization of Atlantic salmon fed the control or microalga feeds for 84 days.

	1C	1L	1H	ANOVA P-value
Survival (%)	100	100	100	
Initial weight (g)	214.5 ± 3.1	213.8 ± 2.7	218.0 ± 2.8	0.76
Final weight (g)	429.0 ± 12.2	420.2 ± 13.3	407.8 ± 12.1	0.50
Weight gain (%)	100.2 ± 5.1	96.3 ± 3.4	86.9 ± 3.2	0.09
SGR (% day^-1^)	0.82 ± 0.03	0.80 ± 0.02	0.74 ± 0.02	0.09
TGC	2.61 ± 0.10	2.54 ± 0.08	2.35 ± 0.08	0.12
FI (% BW day^-1^)	0.68 ± 0.01^b^	0.70 ± 0.01^ab^	0.75 ± 0.02^a^	0.01
FCR	0.81 ± 0.02^bc^	0.86 ± 0.02^b^	1.00 ± 0.06^a^	0.01
PER	2.20 ± 0.06	2.18 ± 0.06	2.03 ± 0.10	0.24

Specific growth rate–SGR. Thermal growth coefficient–TGC. Feed intake–FI. Feed conversion ratio–FCR. Protein efficiency ratio–PER. Different superscripts (a, b, c) in a row indicate statistically significant differences (P < 0.05) among groups. n = 6 tanks, values from 15 fish/tank, mean ± SEM

### Organosomatic indices and hematocrit values

The CF of the study groups ranged between 2.16 and 2.34 (Table 4). Fish of the 1H group had significantly lower CF compared to that of the 1C group. HSI and VSI of the 1L and 1H groups were similar to those of the control group (Table 4). The hematocrit values were significantly different among the three study groups (Table 4). The values of the 1H and 1C differed significantly from each other, while 1L ranked in between.

**Table 4:** Organosomatic indices and hematocrit values of Atlantic salmon fed the control or microalga feeds for 84 days.

	1C	1L	1H	ANOVA P-value
Condition factor	2.34 ± 0.04^a^	2.29 ± 0.04^ab^	2.16 ± 0.04^b^	0.023
Hepatosomatic index	1.27 ± 0.02	1.24 ± 0.01	1.24 ± 0.03	0.563
Viscerosomatic index	8.13 ± 0.09	8.28 ± 0.12	8.33 ± 0.15	0.511
Hematocrit	47 ± 1^b^	50 ± 2^ab^	54 ± 2^a^	0.049

Different superscripts (a, b) in a row indicate statistically significant differences (P < 0.05) among groups. n = 6 tanks, values from 6 fish/tank, mean ± SEM)

### Chemical composition

The proximate composition of whole body and fillet is presented in Table 5. Moisture, protein, lipid and ash contents of the whole body and fillet of the fish from the 3 groups did not vary significantly. However, the lipid content in the whole body of the 3 groups at the end of the feeding trial was lower than that of the initial fish. The lipid content in the fillet was lower compared to that in the whole body.

**Table 5:** Proximate composition (g/100 g dry matter) of Atlantic salmon fed control or microalgae feeds for 84 days.

	Initial	Final		
		1C	1L	1H
*Whole body*				
Moisture	69.2 ± 0.2	68.7 ± 0.2	68.5 ± 0.2	69.2 ± 0.3
Protein	54.7 ± 0.4	55.5 ± 0.5	55.6 ± 0.6	56.3 ± 0.6
Lipid	37.3 ± 0.4	36.4 ± 0.2	36.3 ± 0.5	35.8 ± 0.5
Ash	6.7 ± 0.2	6.0 ± 0.1	6.3 ± 0.2	6.4 ± 0.1
*Fillet*				
Moisture	74.3 ± 0.2	74.4 ± 0.2	74.2 ± 0.1	74.2 ± 0.1
Protein	77.6 ± 0.3	78.8 ± 0.4	79.0 ± 1.0	80.6 ± 0.7
Lipid	18.2 ± 0.4	14.5 ± 0.7	14.8 ± 1.0	14.9 ± 0.4
Ash	5.6 ± 0.1	5.5 ± 0.2	6.1 ± 0.3	5.5 ± 0.1

n = 6 tanks, proximate composition values from pooled samples from 3 fish (for fillet) and 6 fish (for whole body); mean ± SEM

### Protein, lipid and energy retention

Protein, lipid and energy retention in Atlantic salmon from the three study groups are presented in Table 6. Protein retention of the three groups were not significantly different. Lipid and energy retention values of the 1H group were significantly lower compared to the respective values of the 1C group.

**Table 6:** Retention of protein, lipid and energy of Atlantic salmon fed control or microalga feeds for 84 days.

	1C	1L	1H	ANOVA P-value
Protein	39.5 ± 1.4	39.8 ± 0.7	36.2 ± 1.9	0.19
Lipid	66.7 ± 2.1^a^	64.6 ± 4.0^ab^	53.4 ± 3.9^b^	0.04
Energy	46.0 ± 1.4^a^	43.6 ± 1.8^ab^	36.7 ± 2.4^b^	0.01

Values are given as mean ± SEM; n = 6 replicate tanks. Different superscripts (a, b) in a row indicate statistically significant differences (P < 0.05) among groups.

### Digestibility

The ADCs of protein, ash and energy in the three feeds were significantly different (Table 7). The ADC’s of DM and lipid in the alga feeds were significantly different from those of the 1C group.

**Table 7:** Apparent digestibility coefficients (%) of dry matter, protein, ash and energy in the control and microalga feeds.

	1C	1L	1H	P-value
Dry matter	76.0 ± 0.3^a^	71.6 ± 0.4^b^	70.7 ± 0.4^b^	<0.01
Protein	87.9 ± 0.1^a^	85.0 ± 0.4^b^	83.4 ± 0.2^c^	<0.01
Lipid	92.6 ± 0.3^a^	88.6 ± 0.6^b^	87.8 ± 0.3^b^	<0.01
Ash	15.5 ± 1.1^a^	20.8 ± 1.7^b^	30.6 ± 1.2^c^	<0.01
Energy	85.9 ± 0.3^a^	81.5 ± 0.5^b^	79.2 ± 0.3^c^	<0.01

Values are given as mean ± SEM; n = 3, faeces were pooled per tank. Different superscripts (a, b, c) in a row indicate statistically significant differences (P < 0.01) among groups.

### Antioxidant status

**Fig 1:**
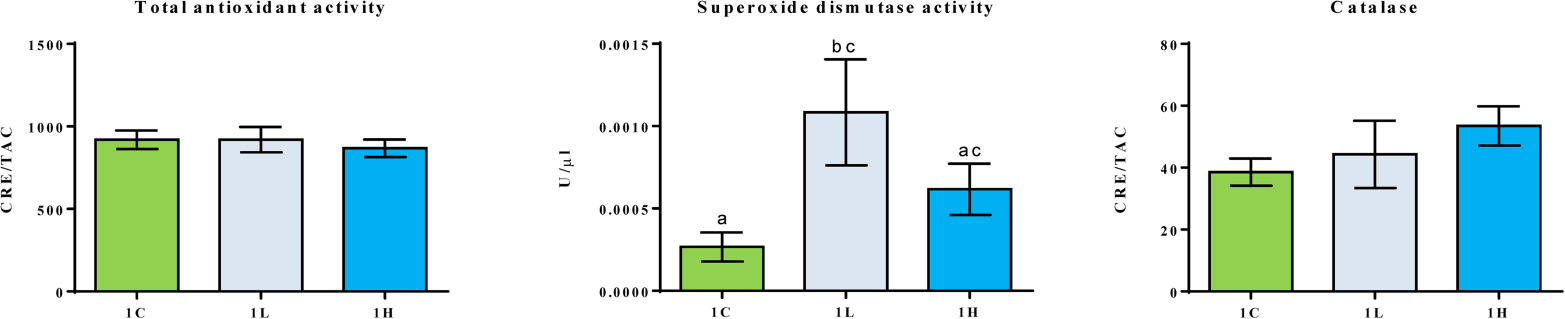
Serum antioxidant capacity of 1C, 1L and 1H groups. Values are expressed as mean ±SEM, n = 6 tanks.Different letters indicate significant differences (P<0.05) between the study groups.

The antioxidant markers such as the TAC and CAT activities in the serum of the 1C, 1L and 1H groups were similar (Fig 1). However, the serum SOD activity of the 1L group was significantly higher than that of the 1C group.

### Intestinal health status

**Fig 2:**
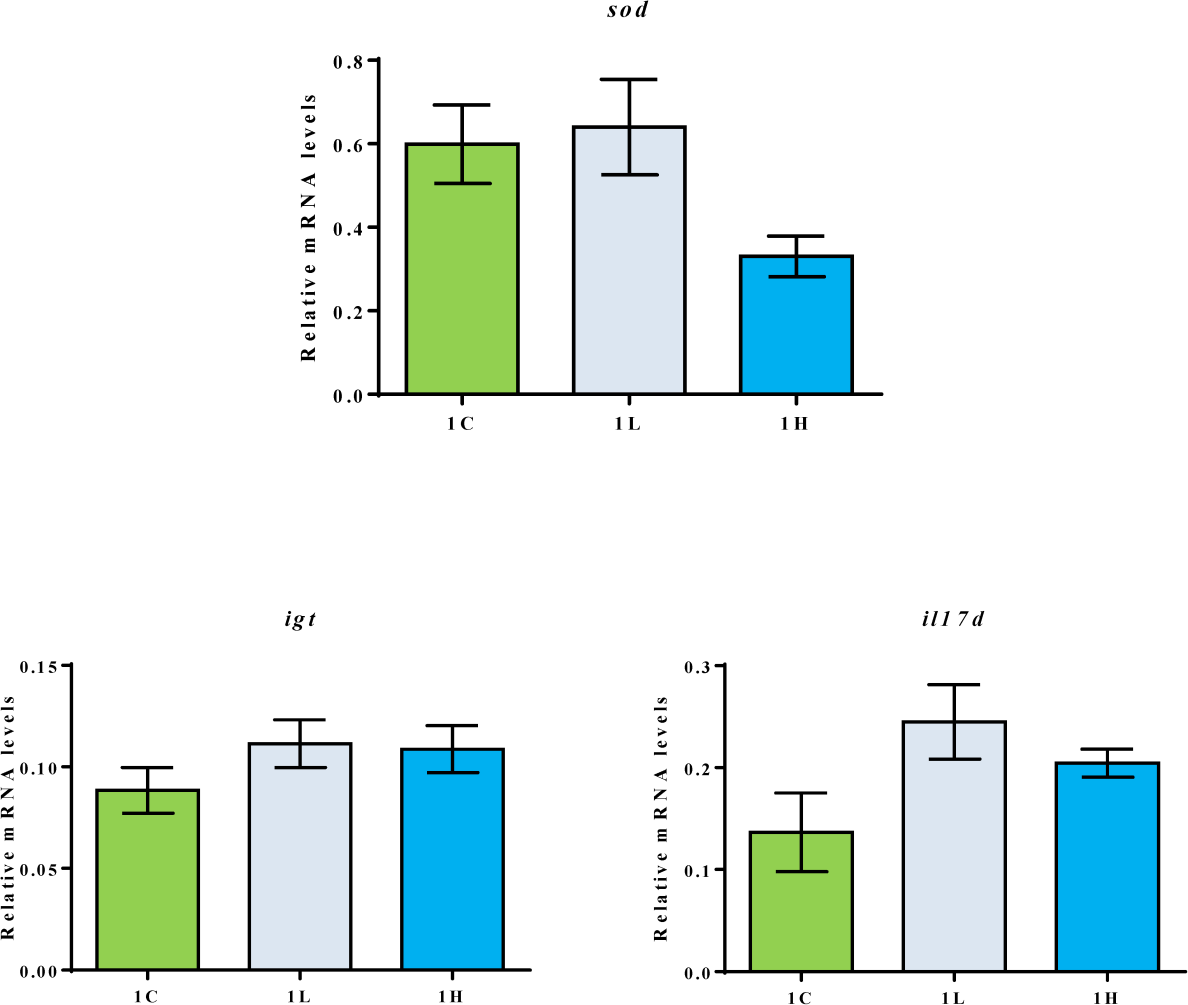
Relative mRNA levels of *sod*, *il17d*, *igt* in the distal intestine of 1C, 1L and 1H groups. The distal intestinal gene expression of the three groups (n = 12 fish) at the end of the 84-days feeding period. Values are presented as mean **±** SEM, n = 6 tanks, 2 fish/tank.

Gene expression: The mRNA level of *il17d* was apparently higher (P<0.1) in the 1L group compared to the level in the control group (Fig 2). In addition, *sod* was apparently higher (P<0.1) in the 1L group compared to the expression in the 1H group. On the other hand, the mRNA levels of *igt* were similar in the study groups. The mRNA levels of *il1b*, *il10*, *tgfb*, *nrf2*, *cath1* and *cath2* were below the detection range.

**Fig 3:**
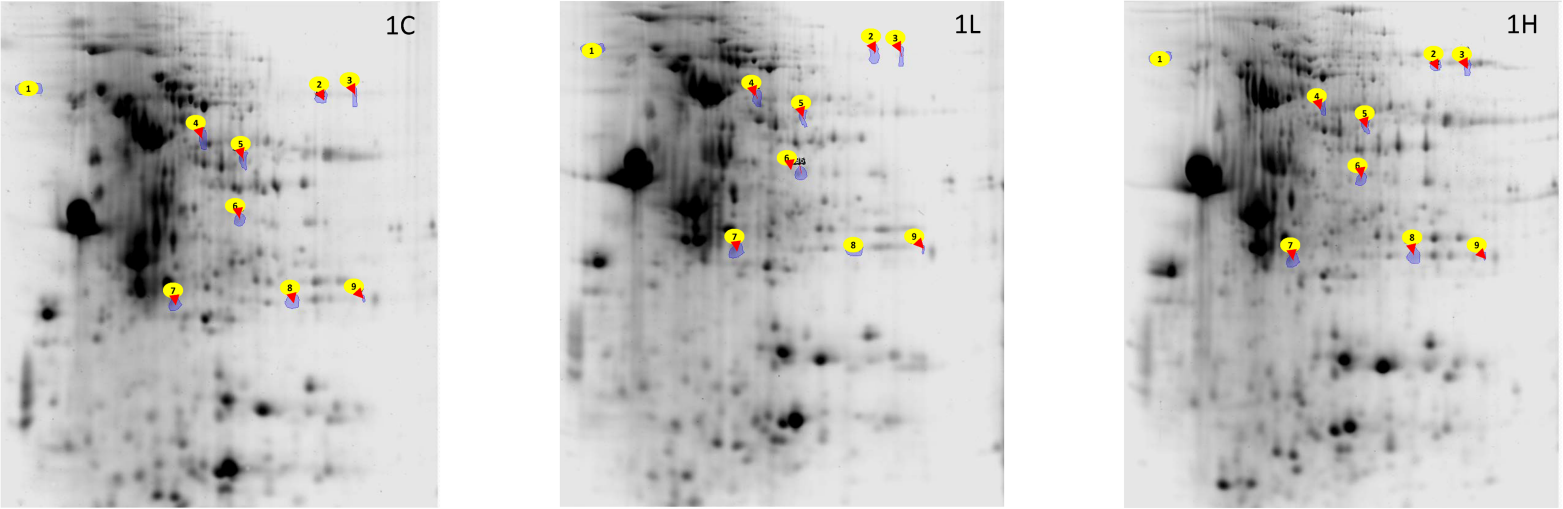
Representative 2-DE gels showing the spots of proteins from the distal intestine of Atlantic salmon. The spots 1–9 corresponds to Ahsg, P5cdh, Dld, Lei, Crt, Ef2, Apoa1-1, Tpi, and Flr, respectively (see Table 8). n = 6 fish/group.

Protein expression: Comparison of the distal intestinal proteome of the fish from the different groups revealed that the expression of 7 proteins were altered by the algal feeding—Delta-1-pyrroline-5-carboxylate dehydrogenase, mitochondrial-like (P5cdh), Dihydrolipoyl dehydrogenase, mitochondrial-like (Dld), Leukocyte elastase inhibitor-like (Lei), Creatine kinase B-type isoform X2 (Crt), Elongation factor 2 (Ef2), Triosephosphate isomerase (Tpi), Flavin reductase (Flr) (Fig 3, S2 Fig, Table 8). On the other hand, the proteins Alpha-2-HS-glycoprotein-like (Ahsg) and Apolipoprotein precursor (Apoa1-1) were significantly underexpressed, 0.5 fold and 0.7-fold respectively, in the 1H group when compared with their expression in the 1L group. Flr was significantly overexpressed (1.7-fold) and Ckt was underexpressed (0.6-fold) in the 1L group when compared to its expression in the other study groups. Tpi was overexpressed (1.8-fold) in the 1L group compared to the expression in the 1C group. Dld (2.5-, 2.8-fold) P5cdh (2.5-, 2.8-fold) were overexpressed in the 1H group compared to the 1C and 1L groups. Ef2 (0.6-fold) and Pfn2 (0.6-fold) were underexpressed in the 1H group compared to the other study groups. Lei (0.5-fold) protein was underexpressed in the 1H group compared to the expression in the 1C group.

**Table 8:** Differentially expressed distal intestinal proteins of Atlantic salmon fed microalga feeds for 84 days.

Spot no.	Protein name	Apparent pI/MW (kDa)	Peptide sequenced^a^
1	Alpha-2-HS-glycoprotein-like, Ahsg	3.0/88.5	**YALNQIDDIK VVTAVEGDCDVVLR ESLFAIMEVGR**
2↑	Delta-1-pyrroline-5-carboxylate dehydrogenase, mitochondrial-like, P5cdh	7.9/88.6	**NEPILGFNEGSPER AADIISGPK TVVQAEIDAAAELIDFFR HAVELESQQPLDSDGSTNTMLYR QVAQNLDVYK SADVQSVVTGTIR STGSIVAQQPFGGAR**
3↑	Dihydrolipoyl dehydrogenase, mitochondrial-like, Dld	8.16/87.8	**NQVTATAEDGSMQVINSK RPDGQIDVAVEAAAGGK NLGLDTVGLELDNR VPSIYAIGDVIAGPMLAHK FPFAANSR**
4↓	Leukocyte elastase inhibitor-like, Lei	6.0/68.8	**TGNVFYSPLSISSALAMVSLGAR ATDNVHVGFNK GAPYALSLANR LYGEQSYQFVETFLGDTK KHYNAELEAVDFK HYNAELEAVDFK NLLAEGVVDHLTR LVLVNAIYFK FKESSTSDALFK ESSTSDALFK NLVEWTRPDMMDTVEVQVGLPK FKLEESLDLK SDFSGMSPNNDLVLSK AFVEVNEEGTEAAGATAAIMMMR**
5↓	Creatine kinase B-type isoform X2, Crt	6.7/60.7	**ILTPAIYER ELLDPIIEDR MSVEALDSLSGDLK GTGGVDTAAVGGTFDISNADR LGFSEVELVQMVVDGVK GQSIDDLMPAQK**
6↓	Elongation factor 2, Ef2	6.6/40.3	**AKPFPDGLAEDIEK EGVLCEENMR TAIVVAETR**
7	Apolipoprotein A-I-1 precursor, Apoa1-1	5.6/29.1	**AALNMYIAQVK SIDLLDDTEYK SIDLLDDTEYKEYK SLAPYTTVFGTQLADATATVR AKIEPVVEEMR IEPVVEEMR VAVNVEETK LMPIVEIVR LMPIVEIVR TLAAPYAEEYKEQMFK**
8↑	Triosephosphate isomerase, Tpi	7.6/28.8	IGVAAQNCYK **GGAFTGEISPAMIK** VVLAYEPVWAIGTGK **ANVSEAVANSVR DVDGFLVGGAALKPEFVDIINAK**
9↑	Flavin reductase, Flr	8.1/29.2	**TMQGQDAVIIILGTR LLPVTEDHDR ESGLDFVAVMPPHIDDNFPLTEK**

^a^Unique peptides are in bold; ↓ indicates underexpression and ↑ indicates overexpression in the algal groups compared to the control group

**Fig 4:**
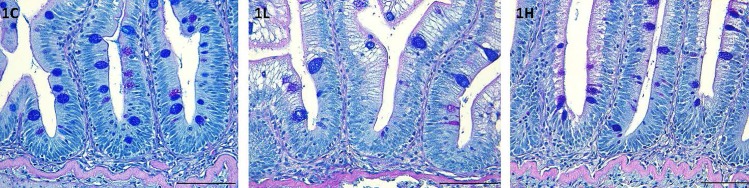
Photomicrographs of the distal intestine of 1C, 1L and 1H groups at the end of the 84-days feeding period. Scale bar: 50μm.

Distal intestinal micromorphology: Feeding the microalga-feeds did not alter the architecture of the distal intestine (Fig 4). Furthermore, there were no signs of inflammation in the intestine.

## Discussion

In the present study, the potential of defatted biomass of the microalga *N*. *oceania* to be an ingredient in the feeds for Atlantic salmon was assessed based on the growth, nutrient digestibility, feed utilization and health parameters.

### Growth and feed performance

The performance of the fish was good throughout the experimental period and SGR was higher than expected, based on growth tables that consider both fish size and water temperature [21]. The SGR values obtained in the present study were slightly lower than those reported earlier [22, 26]. The TGC values in the present experiment were higher than those obtained by Sørensen et al. [15], but were in the same range as reported by Hatlen et al. [26]. Feed conversion rate was in line with studies performed on yeast- or microalga-fed Atlantic salmon of sizes comparable to that used in the present study [26, 27]. The dry matter intake (for each kg wet weight gain) of fish fed the highest inclusion level of defatted *N*. *oceania* was almost 24% greater than that of the control group. Higher feed intake and reduced feed conversion was also reported in other studies investigating microalgae in feeds for Atlantic salmon [28] and European sea bass [29]. The higher feed intake of the 1H group may be a compensation for the slightly lower lipid and energy content in the feeds with 20% alga meal inclusion. The experimental feeds were designed to keep a constant crude protein / energy ratio among the feeds. However, the calculated digestible protein (DP) / digestible energy (DE) values were 25, 24 and 23 for the 1C, 1L and 1H, respectively. These values were within the range 20–24 g DP / DE, which is suggested as optimal for young Atlantic salmon [30]. Increased feed intake to compensate for the low energy content in the feed is a strategy adopted by fish to secure growth [31, 32]. The increased feed intake, noted in our study for Atlantic salmon that received 20% *N*. *oceania*, indicates the palatability of the ingredient. In contrast, other researchers have reported that microalgae may have negative effects on feed intake in fish. Atlantic cod juveniles offered feeds containing 140g/kg mixed biomass of microalgae dominated by *Isochrysis* sp., had reduced feed intake and growth [33]. We cannot make direct comparisons between varieties of algae or even production batches of one particular algae as their biochemical profiles largely depend on the nutrient availability and growing conditions [6].

Digestibility of protein in the fishmeal-based reference feed is in line with other studies [26, 27, 34–37]. However, the overall reduction in digestibility of protein, lipids and energy in the alga feeds may explain the lower weight gain despite the higher feed intake. Some authors have reported similar reductions in nutrient digestibilities upon microalgal feeding [28, 29], while higher microalgal nutrient digestibilities have also been recorded [38]. The digestibility of DM and protein in feeds with 20% *N*. *oceania* is comparable to those obtained by Gong et al. [14], who estimated digestibility of DM and protein of *Nannochloropsis* sp. to be 63% and 72%, respectively. These values are significantly lower than digestibility values of fishmeal fed to salmonids [37, 39, 40], but greater than the protein digestibility (36%) of *Nannochloropsis* in mink [16]. The ADC of lipid and energy showed greater reduction with algae inclusion in the feed compared to the protein. In keeping with these observations, reduced lipid digestibility was reported at 6% inclusion of *Schizohytrium* sp. [27] and 10% inclusion of the yeast *Yarrowia lipolytica* [26]. The reduction in digestibility of lipid and energy in the present study could be due to the high content of complex indigestible cell wall carbohydrates in the microalga [41]. Fish has a limited capacity to digest carbohydrates, in particular the indigestible non-starch polysaccharides [42, 43]. Physical treatment including disruption of cell walls in biomass from *Y*. *lipolytica* caused significantly improved nutrient utilization by Atlantic salmon [44]. Improved ash digestibility noted in the present study is in line with our earlier findings [14]. Interestingly, these findings suggest improved utilization of minerals in the *N*. *oceania*-incorporated feeds.

Protein retention in Atlantic salmon in the present experiment was lower compared to that reported by Hatlen et al. [26]. Energy retention in fish fed the control feed was similar to that in the above publication, but in our case the values decreased with increasing intake of the alga. These findings suggest that protein and energy in defatted *N*. *oceania* are less utilized compared to other single cell protein sources such as the yeast *Y*. *lipolytica* [26].

### Changes in biochemical composition of fish

Neither the whole body, nor fillet proximate composition of Atlantic salmon was affected by the intake of the alga meal. The proximate composition varies with life stages of the fish and is also influenced by endogenous factors such as genetics, size and sex, as well as exogenous factors such as feed composition, feeding frequency and environment [45]. The lipid content of the experimental fish was in the same range as that of similar sized salmon [15, 46]. The higher protein and lower lipid content in fillet compared to whole body are noteworthy results. Another interesting finding is the 19% reduction in lipid content in the fillet of the final samples. This finding was unexpected because of the high correlation between lipid content and size of fish [45]. However, similar results were obtained in other studies too–experiments with Atlantic salmon that were fed *Desmodesmus* [22], trials on Atlantic salmon and common carp fed *Nanofrustulum* [12]. Alne et al. [46] reported that the feeding rate, growth rate and feed utilization of S0 smolt (transferred to sea 8–10 months after hatching) were reduced compared to the performance of S1 smolt that were transferred to sea in the following spring. Thus the CF, muscle fat and retention of energy of S0 smolts were lower compared to S1 smolts. The smolt used in the present experiment was S0 and the drop in lipid content may be explained by physiological changes taking place in the fish related to season. Furthermore, in our case the feed intake of the 1H group was significantly higher than that of the 1C group.

### General physiological status

The organosomatic indices HSI and VSI of the fish fed on feeds with and without alga meal did not vary significantly, and the values were comparable to that reported for Atlantic salmon fed the microalga *Desmodesmus* [22]. *Schizochytrium limacinum* also did not alter the HSI of longfin yellowtail *Seriola rivoliana* [47]. However, previous studies have reported that *Spirulina* feeding can elevate the VSI levels in sturgeon *Acipenser baeri* and parrot fish *Oplegnathus fasciatus* [48, 49].

The increased hematocrit value for the alga-fed groups indicate a positive effect of *Nannochloropsis*. Similar increase was noted in young rockfish, *Sebastes schlegeli*, fed sea mustard (*Undaria pinnatifida*) [50]. A non-significant increase in hematocrit values was also noted in red sea bream, *Pagrus major*, fed *Ulva pertusa* meal [51]. The high oxygen demand to metabolize large amounts of feeds ingested by the 1H group might be the reason for this increase in hematocrit levels. Hematocrit values in fish are tightly associated with environmental parameters such as temperature and oxygen concentration in the rearing water. However, we do not expect the influence of these factors as the fish groups were maintained under identical controlled conditions.

The antioxidant status was determined to understand the alterations in the physiological capacities of the fish. The serum SOD activity in the fish fed on 10% alga-containing feed was higher compared to that in the fish fed on alga-devoid feeds. On the contrary, such an increase was not detected in the fish fed on 20% alga-containing feeds. In our earlier study on *Desmodesmus*, a similar trend in SOD activities was noted [22]. Furthermore, the mRNA level of *sod* was apparently higher in the 10% alga-fed group compared to the level in the 20% alga-fed group. The increased SOD activity may indicate improved antioxidant defence in fish receiving moderate amounts of the alga meal, but this has to be verified through additional investigations.

### Intestinal health condition

In order to confirm the suitability of the alga meal as a feed component, it is necessary to evaluate the intestinal health of the fish through morphological and molecular observations. We examined the expression of selected marker genes related to inflammation and intestinal immune system. Among those studied, the level of the pro-inflammatory gene *il17d* in the 10% alga fed group was apparently higher compared to the level in the control group. However, there were no signs of distal intestinal inflammation in the alga-fed groups in this study, as well as in our previous study [22] using *Desmodesmus*. In inflamed distal intestine of Atlantic salmon, the mRNA levels of *igt* were apparently higher [24], but in the present study the gene expression in the fish from different groups were similar. These findings, taken together, suggest that the microalgal biomass tested do not induce inflammatory reactions in the distal intestine of the fish. Plant ingredients in feeds can trigger inflammatory reactions and aberrations in the distal intestinal structure of Atlantic salmon [52–54]. The n-6 fatty acids in plant-derived feed ingredients can cause intestinal inflammation, and the mid-intestinal folds of Atlantic salmon were shortened by feeding with olive oil, rapeseed oil or soybean oil [55].

To further elucidate the effect of the algal product, we compared the distal intestinal proteomes of salmon that received the different experimental feeds. Nine of the identified proteins were impacted by the alga feeding. This included the protein Apoa1 that has antimicrobial properties in fish [56–58]. The protein was underexpressed in the 1H group when compared to its expression in the 1L group, implying that higher inclusion of the alga meal may affect the defence mechanisms of the fish. Furthermore, we noticed the underexpression of the protein Ahsg in the 1H group. The reduction in levels of the glycoprotein AHSG in the serum of protein-energy-malnourished children was linked to stunted growth, and compromised defence ability [59]. The low Ahsg expression coincided with the lower growth in the 1H group. In our previous report too, feeding *Desmodesmus* led to the underexpression of Ahsg in the distal intestine of Atlantic salmon [22].

Two energy metabolism-related proteins were overexpressed (Flr and Tpi) and one was underexpressed (Ckt) in the distal intestine of the 1L group. The overexpression of Flr and Tpi and the underexpression of Ckt may have benefitted the 1L group. It should be noted that in the 1H group, the lipid and energy retention was significantly lower compared to those of the control group. Dld which is also associated with energy metabolism was significantly overexpressed in the 1H group. On the contrary, lower energy digestibility was associated with the underexpression of Dld when Atlantic salmon was fed on *Desmodesmus* [22].

Two other proteins—Ef2, Lei—were underexpressed and a third one—P5cdh—was overexpressed in the distal intestine of the 1H group. P5CDH is one of the two mitochondrial enzymes that helps the oxidation of proline to glutamate leading to an increase in intracellular reactive oxygen species [60]. Eukaryotic elongation factor 2 (eEF2) mediates the GTP-dependent movement of the ribosome during protein synthesis [61], and increase in oxidative stress has been correlated to decrease in eEF2 [62]. The overexpression of P5cdh and underexpression of Ef2 in the IH group could be pointing to oxidative stress in the fish. This group also had apparently lower SOD activity levels and lower *sod* expression. Leukocyte elastase inhibitor (LEI), also called serpin B1, is a member of the serine protease inhibitors [63]. It is reported that during wound healing LEI expression is increased [64, 65]. Although the Lei-like protein was overexpressed in the distal intestine of the 1H group, we did not observe intestinal damage.

## Conclusions

The results indicate that the defatted microalgae *N*. *oceania* can be used at modest inclusion levels–a level close to 10%–without negative effects on weight gain and specific growth rate and health parameters.

## Supporting information

S1 Table Proximate compostition of defatted microalgae biomass used in feed. (DOCX)

S1 Fig The design of the experimental fish tank. (TIFF)

S2 Fig The volumes of the differentially expressed proteins in the 2-DE gels. * Different letters above the bar graphs indicate statistically significant differences. Values are presented as mean ± SEM.
(TIF)

## Abbreviations

ADC: apparent digestibility coefficients
Ahsg: Alpha-2-HS-glycoprotein-like
AOAC: Association of Official Analytical Chemists
Apoa1-1: Apolipoprotein A-I-1 precursor
CAT: catalase
*cath1* and *cath 2*: *cathelicidin 1* and *2*
CF: Condition factor
Crt: Creatine kinase B-type isoform X2
Dld: Dihydrolipoyl dehydrogenase, mitochondrial-like
DM: dry matter
Ef2: Elongation factor 2
EPA: eicosapentaenoic acid
FCR: Feed conversion rate
FDU: Forsøksdyrutvalget
Flr: Flavin reductase
ForTek: Feed Technology Center
FPC: fillet proximate composition
HSI: hepatosomatic index
igt: *immunoglobulin T*
il10: *interleukin 10*
il17d: *interleukin 17d*
il1b: *interleukin 1b*
ISO: International Organisation for Standardisation
LC-MS/MS: liquid chromatography and tandem mass spectrometry
Lei: Leukocyte elastase inhibitor-like
MS-222: Tricaine methane sulphonate
NMBU: Norwegian University of Life Sciences
nrf2: *nuclear factor erythroid 2-related factor*
NS-EN ISO: Norsk standard
P5cdh: Delta-1-pyrroline-5-carboxylate dehydrogenase, mitochondrial-like
PBS: Phosphate buffered saline
PUFA: Polyunsaturated fatty acids
Ret: Retentions SGR, specific growth rate
SOD: super oxide dismutase
sod1: *superoxide dismutase1*
TAC: total antioxidant capacity
TGC: thermal unit growth coefficient
tgfb: *transforming growth factor beta*
Tpi: Triosephosphate isomerase
VSI: viscerosomatic index
WBC: whole body proximate composition
