# Supplementary material for: Nannochloropsis oceania-derived defatted meal as an alternative to fishmeal in Atlantic salmon feeds
Source: PLoS One. 2017 Jul 13;12(7):e0179907. doi: 10.1371/journal.pone.0179907 (PMC5509142; doi:10.1371/journal.pone.0179907)
Supplement: S1 Table — (DOCX) [file pone.0179907.s001.docx]

**S1 Table****. Proximate composition of defatted microalgae biomass used in feed.**

| **Content^1^** | |
| --- | --- |
| Moisture | 2.2 |
| In dry matter (g/100g) |  |
| Crude protein | 43.0 |
| Crude lipid | 2.5 |
| Ash | 23.5 |
| Carbohydrate^2^ | 28.8 |
| Energy KJ g ^-1^ | 19.0 |

^1^ Analyses of 4 samples of defatted algae biomass.

^2^Carbohydrates were calculated by differences (100-moisture-crude protein-crude lipid-ash)
