# Supplementary figures and images for: Nannochloropsis oceania-derived defatted meal as an alternative to fishmeal in Atlantic salmon feeds
Source: PLoS One. 2017 Jul 13;12(7):e0179907. doi: 10.1371/journal.pone.0179907 (PMC5509142; doi:10.1371/journal.pone.0179907)

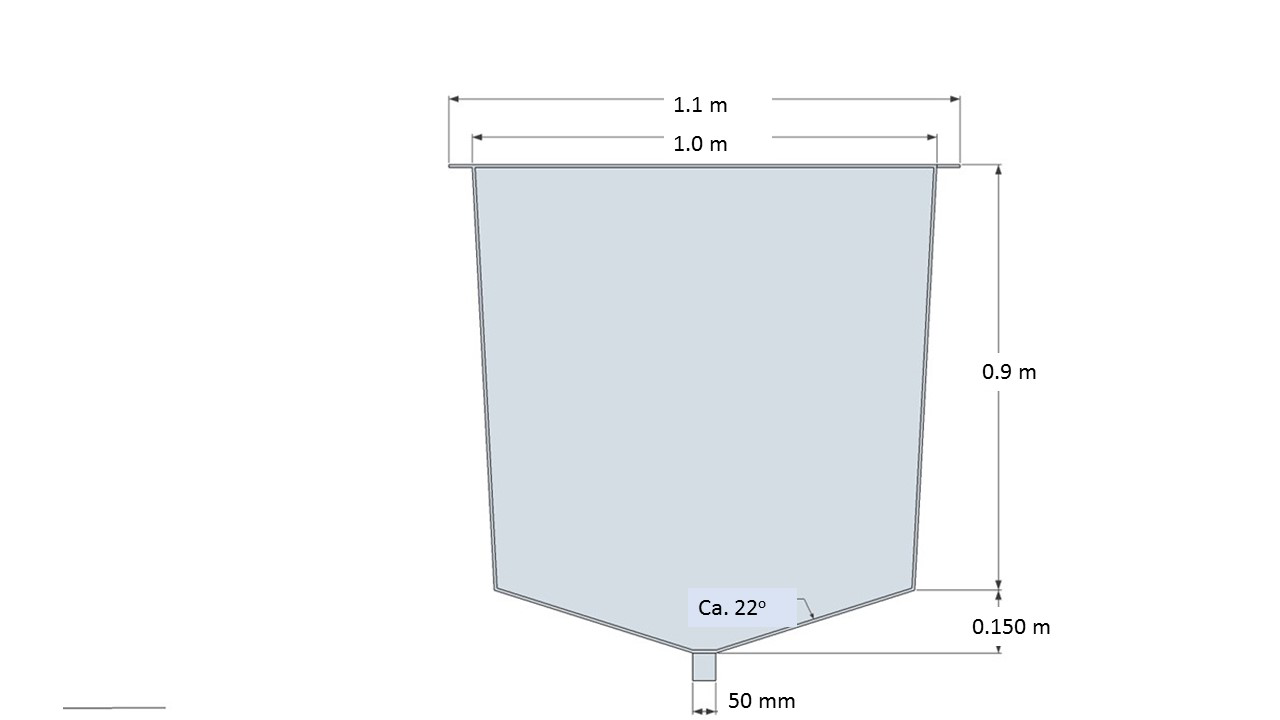

Supplement: S1 Fig — (TIFF) [file pone.0179907.s002.tiff]

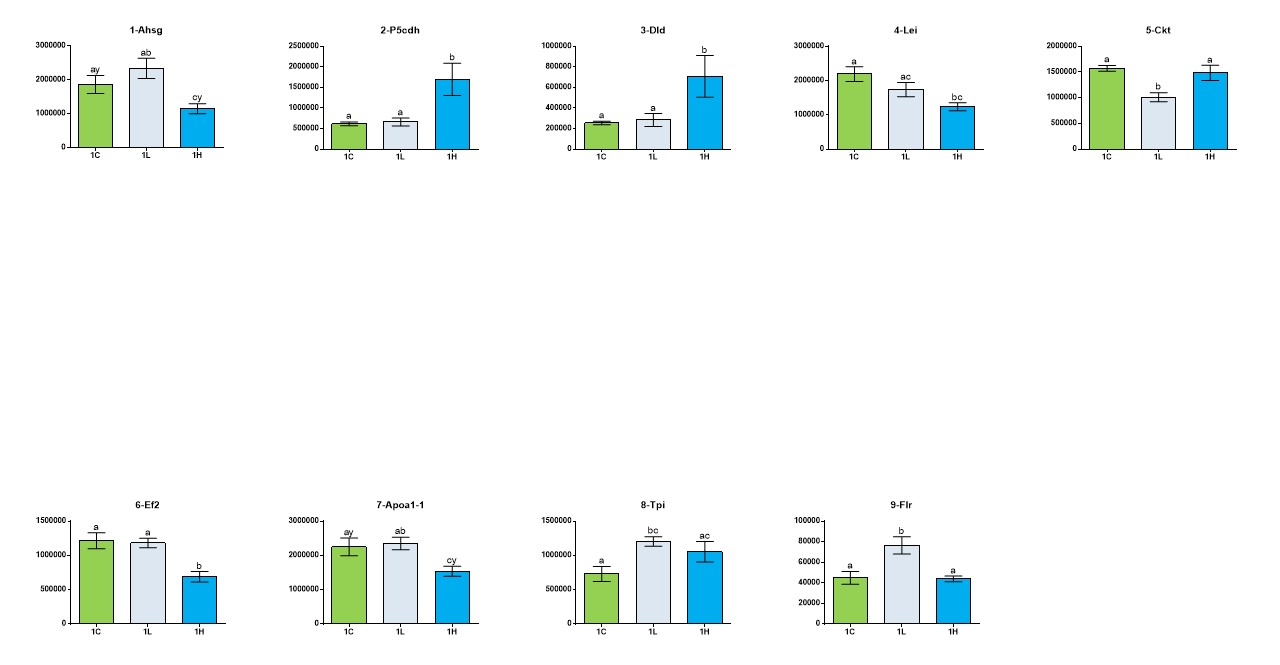

Supplement: S2 Fig — * Different letters above the bar graphs indicate statistically significant differences. Values are presented as mean ± SEM. (TIF) [file pone.0179907.s003.tif]
